# Hepatitis C Virus Elimination Using Direct Acting Antivirals after the Radical Cure of Hepatocellular Carcinoma Suppresses the Recurrence of the Cancer

**DOI:** 10.3390/cancers14092295

**Published:** 2022-05-04

**Authors:** Ryoko Kuromatsu, Tatsuya Ide, Shusuke Okamura, Yu Noda, Naoki Kamachi, Masahito Nakano, Tomotake Shirono, Shigeo Shimose, Hideki Iwamoto, Reiichiro Kuwahara, Teruko Arinaga-Hino, Takashi Niizeki, Yuki Zaizen, Hiroshi Takaki, Miki Shirachi, Hironori Koga, Takuji Torimura

**Affiliations:** 1 Division of Gastroenterology, Department of Medicine, Kurume University School of Medicine, Kurume 830-0011, Japan; ide@med.kurume-u.ac.jp (T.I.); okamura_shyuusuke@kurume-u.ac.jp (S.O.); noda_yuu@med.kurume-u.ac.jp (Y.N.); kamachi_naoki@med.kurume-u.ac.jp (N.K.); nakano_masahito@kurume-u.ac.jp (M.N.); shirono_tomotake@med.kurume-u.ac.jp (T.S.); shimose_shigeo@med.kurume-u.ac.jp (S.S.); iwamoto_hideki@med.kurume-u.ac.jp (H.I.); ray@med.kurume-u.ac.jp (R.K.); terukoh@med.kurume-u.ac.jp (T.A.-H.); niizeki_takashi@kurume-u.ac.jp (T.N.); hirokoga@med.kurume-u.ac.jp (H.K.); tori@med.kurume-u.ac.jp (T.T.); 2 Department of Gastroenterology, Saga Central Hospital, Saga 849-8522, Japan; zaizen_yuki@med.kurume-u.ac.jp; 3 Department of Gastroenterology, Kumamoto Central Hospital, Kumamoto 869-1102, Japan; takaki_hiroshi@kurume-u.ac.jp; 4 Department of Gastroenterology, Chikugo City Hospital, Fukuoka 833-0041, Japan; chikugo-hpdr@mx21.tiki.ne.jp

**Keywords:** hepatocellular carcinoma, hepatitis C virus, direct-acting antiviral, recurrence, radiofrequency ablation, hepatic resection, propensity score matching

## Abstract

**Simple Summary:**

In patients with hepatitis C virus-related liver disease, direct-acting antivirals (DAAs) suppress the development of hepatocellular carcinoma (HCC). However, it is unclear whether their use after curative HCC treatment suppresses its recurrence in patients with hepatitis C virus-related liver disease. We retrospectively evaluated the inhibitory effect of DAAs on HCC recurrence using propensity score matching. Both the first and second HCC recurrence rates in the DAA-treated group were lower than those in the non-DAA-treated group, suggesting that the inhibitory effect of DAA therapy on HCC recurrence is sustained.

**Abstract:**

It remains unclear whether hepatocellular carcinoma (HCC) recurrence in hepatitis C virus (HCV)-infected patients can be suppressed by the elimination of the virus using direct-acting antivirals (DAAs) after radical HCC treatment. We evaluated the sustained inhibitory effect of DAAs on HCC recurrence after curative treatment. This multicenter retrospective study included 190 HCV-positive patients after radical treatment for early-stage HCC. Patients were classified into the DAA treatment group (n = 70) and the non-DAA treatment group (n = 120) after HCC treatment. After propensity score matching (PSM), 112 patients were assessed for first and second recurrences using the Kaplan–Meier method and analyzed using a log-rank test. The first recurrence rates at 1 and 3 years were 3.6% and 42.1% in the DAA treatment group and 21.7% and 61.9% in the non-DAA treatment group, respectively (*p* = 0.0026). Among 85 patients who received radical treatment, the second recurrence rate at 3 years was 2.2% in the DAA treatment group and 33.9% in the non-DAA treatment group (*p* = 0.0128). In HCV-positive patients with early-stage HCC, the first and second recurrences were suppressed by DAA therapy after radical treatment, suggesting that the inhibitory effect of DAA therapy on HCC recurrence was sustained.

## 1. Introduction

Recent studies report approximately 95% efficacy of direct-acting antiviral (DAA) therapy in the elimination of the virus in hepatitis C virus (HCV)-related chronic liver disease (CLD) [1,2], as well as improvement of liver fibrosis [3,4]. Furthermore, the incidence of HCC has been reported to decrease in patients in whom the virus had been eliminated by DAA therapy [5,6,7]. However, it remains unclear whether DAA therapy after radical treatment of HCC inhibits its recurrence. Many studies have reported that DAA treatment increases the survival rate but does not change the recurrence rate of HCC [8,9,10]. In contrast, DAA therapy after HCC treatment has been reported to cause rapid HCC recurrence [11]. Few reports have shown a decrease in recurrence rates [12,13,14]. Even if HCC is detected early and subjected to radical treatment, new cancerous nodules may later develop at other sites; this is known as multicentric recurrence [15]. Among HCC-causing background liver diseases, such as hepatitis B virus- or HCV-related CLD, alcoholic liver disease, non-alcoholic steatohepatitis, and autoimmune liver diseases, the highest incidence of HCC can be found in those with HCV-related CLD [16,17]. In addition, the incidence rate of multicentricity is highest in patients with HCV-related CLD, as compared to patients with hepatitis B virus-related CLD or non-B/non-C liver disease [18,19]. Suppression of multicentric recurrence is important for the prognosis and prolongation of survival in patients with HCV-positive HCC. In this study, using retrospective multicenter data, the rates of first and second recurrences were compared in patients who had achieved sustained virological response (SVR) upon receiving DAA treatment and in patients who had not been treated with DAA after radical treatment of HCC. Based on these findings, our study examined (1) whether DAA therapy early after radical treatment of HCC had an inhibitory effect on recurrence and (2) whether the inhibitory effect on HCC was sustained.

## 2. Materials and Methods

### 2.1. Patient Enrollment and Study Design

Patients with HCC within Barcelona Clinic Liver Cancer (BCLC) stage A [20], who were treated radically with hepatectomy or radiofrequency ablation between January 2010 and December 2017 at Kurume University Hospital (Kurume, Japan), and from affiliated institutions, namely Kurume University Medical Center (Kurume, Japan), Kurume Central Hospital (Kurume, Japan), Yame General Hospital (Yame, Japan), Saga Central Hospital (Saga, Japan), Kumamoto Central Hospital (Kumamoto, Japan), Chikugo City Hospital (Chikugo, Japan), Yanagawa Hospital (Yanagawa, Japan), and Yasumoto Hospital (Kurume, Japan), were retrospectively included in this study. The final observation was conducted in October 2020. The exclusion criteria were as follows: death within 1 year after radical treatment of HCC and achievement of SVR upon receiving interferon (IFN) and DAA therapy before HCC treatment or after treatment of HCC recurrence. Patients who did not wish to be treated with DAA because of high age, financial problems, inactivity of hepatitis, or concerns about adverse effects were included in the untreated group. All patients of the non-DAA-treated group were confirmed positive for HCV-RNA.

Seventy patients started receiving DAA within 1 year after HCC treatment and achieved SVR and were classified into the DAA-treated group, and 120 cases were classified into the non-DAA-treated group. The DAA-treated and non-DAA-treated groups, following propensity score matching (PSM) to reduce confounding, were compared on the basis of recurrence rates and re-recurrence rates (Supplementary Figure S1), and factors involved in recurrence and re-recurrence were analyzed.

The study protocol conformed to the principles of the Declaration of Helsinki and was approved by the Ethics Committee of Kurume University (approval number: 14178). Informed consent was obtained from the patients through a specific from on the website. Patients who denied consent were excluded.

### 2.2. Diagnosis, Treatment, and Assessment of Treatment Effect in HCC and Follow-Up Schedule

HCC was diagnosed in accordance with standard guideline [21]. Whether HCC was within BCLC stage A was determined using liver tumor biopsy and/or at least two of the following imaging modalities: dynamic computed tomography, magnetic resonance imaging, and contrast-enhanced ultrasonography. Radical treatment of HCC was done by hepatectomy, or radiofrequency ablation conducted percutaneously, laparoscopically, or by laparotomy. The treatment effect was determined 1 to 3 months after treatment by conducting dynamic computed tomography or magnetic resonance imaging and using the modified response evaluation criteria in solid tumors guideline [22]. Post-HCC treatment follow-up consisted of abdominal ultrasonography and laboratory tests, including tumor markers, every 2–4 months and dynamic computed tomography or magnetic resonance imaging every 6 months.

### 2.3. Treatment Using DAA

DAA treatment was initiated within 1 year of HCC therapy. Dynamic computed tomography or magnetic resonance imaging was performed to confirm the absence of HCC recurrence within 3 months before the initiation of DAA treatment. DAA treatment was administered for 8–24 weeks using medication regimens selected according to the genotype: daclatasvir and asunaprevir, sofosbuvir and ribavirin, ledipasvir/sofosbuvir, and lecaprevir/pibrentasvir. SVR was defined as the absence of detectable HCV RNA at 24 weeks after the end of treatment.

### 2.4. Collection of Clinical and Laboratory Data

The following data were collected: age, sex, hepatitis B surface antigen, alcohol consumption history, presence or absence of diabetes mellitus, clinical data (including history of prior interferon (IFN) therapy), platelet count, albumin, aspartate aminotransferase (AST), alanine aminotransferase (ALT), alpha-fetoprotein (AFP), des-gamma-carboxy prothrombin (DCP), tumor diameter, and the number of tumors at diagnosis of HCC. Child–Pugh (CP) class, FIB-4 index, and modified albumin–bilirubin (mALBI) score [23,24] were calculated from these data.

### 2.5. PSM Analysis

To reduce confounders, a PSM analysis was used. Propensity scores were estimated using logistic regression models in which the following were used as covariates: age; sex; presence of hepatitis B surface antigen; alcohol consumption history; presence or absence of diabetes; history of IFN therapy; platelet count; albumin; AST, ALT, AFP, and DCP levels at the time of HCC diagnosis; tumor diameter; number of tumors; CP class; FIB-4 index; and modified ALBI score. The cut-off values for age; platelet count; and AST, ALT, and albumin levels were determined based on receiver operating characteristics, and reference values were used as cut-off values for AFP and DCP levels. Fifty-six pairs of patients were selected using the 1:1 nearest neighbor matching algorithm with an optimal caliper of 0.2, without replacement.

### 2.6. Statistical Analysis

The number of cases or the median value (range) is mentioned in the data. All statistical analyses were performed using JMP Pro version 15 (SAS Institute Inc., Cary, NC, USA). To compare factors between the two groups before and after PSM, the chi-square test was used for categorical variables and the Mann–Whitney U test (non-normal distribution data) and Student’s t-test (normal distribution data) were used for continuous variables. Statistical significance was set at *p* < 0.05. The recurrence and second recurrence rates were determined using the Kaplan–Meier method, and the differences between the two groups were analyzed using the log-rank test. The factors contributing to recurrence and second recurrence were examined using the Cox proportional hazards model.

## 3. Results

### 3.1. Background Factors

Table 1 shows the clinical characteristics of the DAA-treated and untreated groups. No significant differences were found in terms of sex, alcohol consumption, presence or absence of diabetes, hepatitis B surface antigen positivity, tumor diameter, CP class indicating liver cirrhosis or liver fibrosis, FIB-4 index, and platelet count. Age was higher (*p* = 0.0002) and ALT levels (*p* = 0.0114) were lower in the non-DAA-treated group, and significantly more patients had a history of IFN therapy in the DAA-treated group (*p* = 0.0156). The serum AFP levels showed no significant difference. Serum DCP levels were significantly higher in the non-DAA-treated group (*p* = 0.0156); however, the median levels were below the reference values in both groups. There was no significant difference in the observation period from the time of HCC treatment between the two groups.

As indicated in Table 2, the comparison of 112 cases after PSM showed no significant differences in DCP and ALT levels which had shown a significant difference before PSM. The non-DAA-treated group had significantly more patients who were older and/or untreated with IFN. No significant differences were found in the duration of the observation.

### 3.2. Comparison of Recurrence Rates in the DAA-Treated Group and Non-DAA-Treated Group after PSM

**Figure 1:**
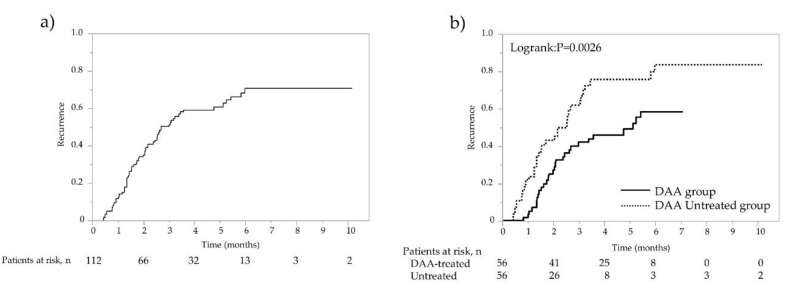
Recurrence rate after propensity score matching (PSM) analysis. (**a**) Overall recurrence rate of patients after curative treatment for HCC. (**b**) Recurrence rates of patients with and without DAA treatment following HCC curative treatment. Solid line represents DAA therapy group; dotted line represents non-DAA-treated group.

**Figure 2:**
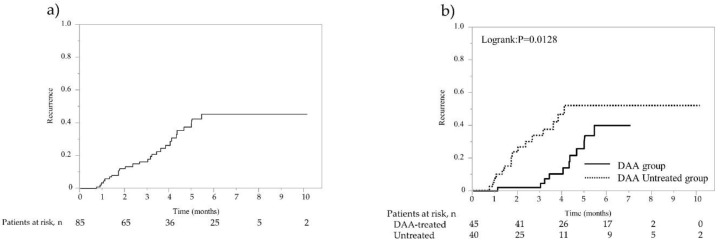
Second recurrence rate after propensity score matching (PSM) analysis. (**a**) Overall second recurrence rate of 85 patients with curative treatment for initial and first recurrent HCC. (**b**) Second recurrence rates of 85 patients with curative treatment for initial and first recurrent HCC. Solid line represents DAA therapy group; dotted line represents non-DAA-treated group.

The overall recurrence rates at 1, 2, and 3 years were 12.6%, 35.2%, and 51.6% (13.7/100 person-years at risk), respectively (Figure 1a). In the DAA-treated group, recurrence was found in 29 cases with a median observation period of 57.8 months, whereas in the non-DAA-treated group, recurrence was found in 38 cases with a median period of 43.3 months. The 1-year, 2-year, 3-year, 4-year, and 5-year recurrence rates were 3.6%, 27.2%, 42.1%, 46.2%, and 49.2% (11.5/100 person-years at risk), respectively, in the DAA-treated group, which were significantly lower than the 21.7%, 42.9%, 61.9%, 75.5%, and 75.5% (16.1/100 person-years at risk) in the non-DAA-treated group (*p* = 0.0026; Figure 1b). Recurrence was observed in 67 of 112 patients, and among these, radical treatment was performed in 40 patients. In the 85 cases including both recurrence-free cases and cases in which curative treatment was performed after recurrence, second recurrence was found in 11 cases with a median observation period of 57.8 months in the DAA-treated group, whereas recurrence was found in 16 cases with the median period of 46.7 months in the non-DAA-treated group. The rates of overall second recurrences at 1, 2, and 3 years were 3.5%, 12.2%, and 16.5% (7.3/100 person-years at risk), namely 0%, 2.2%, and 2.2% (5.4/100 person-years at risk) for the 45 cases in the DAA-treated group and 7.5%, 23.7%, and 33.9% (9.5/100 person-years at risk) for the 40 cases in the non-DAA-treated group, respectively, showing that the rates of second recurrences were significantly lower in the DAA-treated group (*p* = 0.0128; Figure 2a,b).

The results were similar to those of the comparison of recurrence rates in the DAA-treated and non-DAA-treated groups before PSM (Supplementary Figure S2).

### 3.3. Factors Contributing to Recurrence-Free and Second-Recurrence-Free Survival after PSM

Univariate analysis of factors involved in recurrence-free survival in 112 cases after PSM showed that the following were significant: treatment with DAAs (*p* = 0.0032), presence of a single tumor (*p* = 0.0030), and AFP levels of 10 ng/mL or lower (*p* = 0.0304). Multivariate analysis showed that treatment with DAAs (*p* = 0.0034) and the presence of a single tumor (*p* = 0.0344) were significant factors (Table 3). A significant factor involved in the second-recurrence-free survival in 85 cases was only DAAs (*p* = 0.0394) in multivariate analysis (Table 4).

### 3.4. Factors Contributing to Recurrence-Free and Second-Recurrence-Free Survival in the Daa-Treated Group after PSM

For the 56 patients in the DAA-treated group, no significant factors contributing to recurrence-free survival were found in either univariate or multivariate analysis. The presence of a single tumor tended to contribute to recurrence in multivariate analysis (*p* = 0.0731). In contrast, the only significant factor contributing to second recurrence in both univariate (*p* = 0.0051) and multivariate analyses (*p* = 0.0016)) was the presence of a single tumor (Table 5, Supplementary Figure S3).

## 4. Discussion

The purpose of this study was to examine the effect of DAA therapy on tumor recurrence after radical treatment of BCLC stage A HCC. Our findings showed that DAA therapy after radical treatment of HCC suppressed both recurrence and second recurrence. The suppressive effect on recurrence was particularly prominent in patients with a single tumor.

With the former IFN therapy, HCC development was suppressed when SVR was achieved [25,26]. Furthermore, after radical treatment of HCC, tumor recurrence is suppressed by achieving SVR with IFN or long-term administration of low doses of IFN without SVR [27,28,29,30]. In contrast, although a number of studies have reported that achievement of SVR upon receiving DAA therapy inhibited the development of HCC [5,6,7], it remains unclear whether using DAA after HCC treatment suppresses recurrence. Reig et al. previously reported that, in some cases, the use of DAAs after HCC treatment was found to cause early recurrence [11]. Singal et al. refuted the aforementioned by stating in a retrospective study of 793 HCC patients that a comparison between the DAA-treated group and the untreated group showed no difference in recurrence rates after HCC treatment [31]. Kinoshita et al. reported no significant difference in the HCC recurrence rate between IFN and DAA [32]. In a prospective study, Cabibbo et al. reported no significant difference in recurrence rates between DAA-treated patients and untreated controls [8]). Several studies have reported that recurrence rates were reduced by the use of DAAs after treatment for HCC [12,13,14,33,34]. However, the short median observation period has been a major issue in reports published to date. Other reasons are that the studies included cases in which DAA therapy was carried out after treatment of a recurrent HCC rather than after treatment of initial HCC, the cases examined in those studies included patients who had received treatment other than radical treatment, and the duration of the period from the radical treatment of HCC until the administration of DAA therapy was unclear.

Periodic observation after radical treatment for HCC was performed using almost the same method in all patients. The median observation period after HCC treatment was 56 months, which was longer than that in previous reports. In addition, confounding factors were reduced using PSM analysis. The results showed that the recurrence rate was reduced by DAA therapy after radical treatment of HCC. It was suggested that the decrease in HCC recurrence rate was associated with suppression of inflammation and fibrosis by DAA therapy, similar to the reduced HCC incidence after IFN and DAA therapy [35,36].

We further confirmed that DAA therapy prevented second recurrences. Although suppression of first recurrences by long-term low-dose IFN treatment without achievement of SVR after radical treatment of HCC is unclear, suppression of second and third recurrences has been reported [28,37,38]. No previous report has stated that DAA therapy after radical cure of HCC suppressed the first and second recurrences, and the results described above suggested that similar to IFN therapy, the suppressive effect of DAA treatment on HCC was persistent. The findings of our present study further clarify the need for DAA treatment after the radical cure of HCC. In the DAA-treated group, the presence of the first cancer as a single tumor is a significant factor contributing to recurrence-free and secondary-recurrence-free survival rates. The results may be meaningful in determining the intervals between and duration of surveillance.

Alcohol history, liver fibrosis, and diabetes have been reported as factors contributing to hepatic carcinogenesis after SVR [39]. However, in the present study, alcohol history, diabetes, platelet count, and ALBI grade (an indicator of liver fibrosis) did not contribute to recurrence or second recurrence in patients with HCC after SVR. This may be related to the shorter follow-up period after SVR in the present study compared to that of the previous one examining the risk of HCC after DAA treatment.

In the present study, the observation period was three years longer in the non-DAA-treated group than in the DAA-treated group. Although the same observation period is desirable, we added the 3-year period of 2010–2012 to the observation period of the untreated group to increase the number of patients for statistical robustness to minimize potential lead time bias. It seemed valid to prolong the observation period for the following reasons: during these three years, the quality of both the diagnostic modalities, including MRI and contrast-enhanced ultrasonography, and the radical treatment, including radiofrequency ablation therapy, were considered unchanged. Furthermore, there were no significant differences in patient backgrounds between the 70 patients in 2010–2012 and the 120 patients in 2013–2017.

This study had several limitations. First, it was a retrospective study with a small sample size. A prospective randomized study or cohort study of treatment would be desirable, but given the reported improvement in hepatic reserve and prognosis by DAA treatment, it would be difficult to accumulate untreated cases in the future. The second limitation is the inadequate investigation of risk factors for liver cancer other than HCV. In addition to diabetes and alcohol drinking, which were examined in this study, obesity and diabetes medications such as insulin should be considered.

## 5. Conclusions

In conclusion, first and second recurrences were suppressed by DAA therapy after radical treatment for HCC in patients with HCV-related HCC within BCLC stage A, suggesting that the inhibitory effect of DAA therapy on HCC recurrence was sustained.

## Figures and Tables

**Table 1:** Patient characteristics before propensity score matching.

Characteristic	All Patients (n = 190)	Patients Treated with DAAs (n = 70)	Untreated Patients	*p*-Value
			(n = 120)	
Age	72 (44–93)	71 (56–88)	77 (54–93)	0.0002
Sex (Male/Female)	101/89	41/29	60/60	0.2534
HBsAg (+/−)	3/187	1/69	2/118	0.8989
Alcohol history (+/−/unknown)	28/156/6	12/56/2	16/100/4	0.4824
Diabetes mellitus (+/−/unknown)	51/137/2	19/50/1	32/87/1	0.9236
IFN treatment history (+/−)	37/153	20/50	17/103	0.0156
Child–Pugh score (5/6/7)	94/66/30	36/24/10	58/42/20	0.8821
ALBI score	−2.309 (−3.296 to −1.056)	−2.340 (−3.296 to −1.056)	−2.275 (−3.216 to −1.155)	0.5778
FIB-4 index	5.04 (1.03–27.64)	4.93 (1.03–18.67)	5.19 (1.10–27.64)	0.5969
Platelet count (×10^4^/mm^3^)	10.8 (2.5–37)	10.7 (2.5–36.1)	10.8 (3.1–37)	0.9028
Albumin (g/dL)	3.62 (2.44–4.7)	3.65 (2.44–4.6)	3.59 (2.59–4.7)	0.666
AST (IU/L)	51 (18–169)	52 (19–169)	48 (18–141)	0.1599
ALT (IU/L)	40.5 (6–224)	46.5 (11–224)	37 (6–124)	0.0114
Tumor size (mm)	17 (8–45)	16 (8–42)	18 (9–45)	0.1288
Tumor numbers (1/2/3)	139/35/16	1955/9/6	84/26/10	0.3151
AFP (ng/mL)	11.5 (1.2–8461)	9.0 (1.5–553)	13.6 (1.2–8461)	0.4003
DCP (mAU/mL)	24 (3–1657)	22 (3.9–246)	27.5 (3–1657)	0.0199
HCC treatment (Resection/RFA)	52/138	18/52	34/86	0.6961
Observation Period (months)	48.9 (12.2–121.8)	57.2 (14.4–84.6)	45.0 (12.2–121.8)	0.3071

Data are expressed as median (range) or number. Abbreviations: DAA, direct-acting antiviral; HBsAg, hepatitis B surface antigen; ALBI score, albumin–bilirubin score; AST, aspartate aminotransferase; ALT, alanine aminotransferase; AFP, α-fetoprotein; DCP, des-γ-carboxy prothrombin; HCC, hepatocellular carcinoma; RFA, radiofrequency ablation.

**Table 2:** Patient characteristics after propensity score matching.

Characteristic	All Patients (n = 112)	SVR with DAAs (n = 56)	Non-DAA (n = 56)	*p*-Value
Age	73 (55–93)	69.5 (56–85)	76.5 (55–93)	0.0002
Sex (Male/Female)	56/56	32/24	24/32	0.1306
HBsAg (+/−)	1/111	1/55	0/56	0.3151
Alcohol history (+/−/unknown)	14/95/3	10/45/1	4/50/2	0.0928
Diabetes mellitus (+/−/unknown)	34/77/1	16/40/0	18/37/1	0.6349
IFN treatment history (+/−)	26/86	18/38	8/48	0.0252
Child–Pugh score (5/6/7)	52/43/17	29/20/7	23/23/10	0.4889
ALBI score	−2.276 (−3.295 to −1.056)	−2.321 (−3.295 to −1.056)	−2.240 (−3.216 to −1.329)	0.4885
FIB-4 index	5.42 (1.03–27.64)	4.89 (1.03–18.67)	6.26 (1.61–27.64)	0.1067
Platelet count (×10^4^/mm^3^)	10.2 (2.5–36.1)	10.7 (2.5–36.1)	9.8 (3.1–23.8)	0.1669
Albumin (g/dL)	3.59 (2.44–4.6)	3.64 (2.44–4.6)	3.48 (2.77–4.6)	0.5482
AST (IU/L)	52 (19–169)	53 (19–169)	52 (19–141)	0.8376
ALT (IU/L)	43 (10–224)	45.5 (11–224)	41 (10–124)	0.2791
Tumor size (mm)	17 (8–45)	15.5 (8–42)	18 (10–45)	0.3952
Tumor numbers (1/2/3)	80/23/9	1944/7/5	36/16/4	0.109
AFP (ng/mL)	13.6 (1.2–553)	13.4 (1.5–553)	13.8 (1.2–258)	0.2827
DCP (mAU/mL)	22 (3–1657)	23 (3.9–246)	20.5 (3–1657)	0.2902
HCC treatment (Resection/RFA)	25/87	15/41	10/46	0.2565
Observation Period (months)	50.5 (14.4–121.8)	57.8 (14.4–84.6)	45.3 (15.8–121.8)	0.4241

Data are expressed as median (range) or number. Abbreviations: DAA, direct-acting antiviral; HBsAg, hepatitis B surface antigen; ALBI score, albumin–bilirubin score; AST, aspartate aminotransferase; ALT, alanine aminotransferase; AFP, α-fetoprotein; DCP, des-γ-carboxy prothrombin; HCC, hepatocellular carcinoma; RFA, radiofrequency ablation.

**Table 3:** Univariate and multivariate analyses of HCC recurrence-free survival after PSM.

Variable	Category	Univariate Analysis		Multivariate Analysis	
		HR (95% CI)	*p*-Value	HR (95% CI)	*p*-Value
Age	≤74	1 1.265 (0.78–2.04)	0.3375		
	>74				
Sex	Male Female	1 1.149 (0.71–1.86)	0.5711		
HBsAg	Positive Negative	5.4571 × 10^−9^ (0–0) 1	0.9989		
DAA	Untreated Treated	1 0.480 (0.29–0.78)	0.0032	1 0.463 (0.28–0.77)	0.0034
Alcohol history	+ −	0.850 (0.41–1.78) 1	0.6665		
Diabetes mellitus	+ −	1.435 (0.86–2.38) 1	0.1636	1.277 (0.74–2.21) 1	0.3807
IFN	+ −	0.706 (0.40–1.26) 1	0.2376		
treatment history					
Child–Pugh	5 6–7	0.764 (0.47–1.24) 1	0.2747		
ALBI score	1–2a 2b–3	0.730 (0.45–1.18) 1	0.1985	1.260 (0.70–2.29) 1	0.4417
FIB-4 index	≤3.25 >3.25	0.649 (0.31–1.36) 1	0.2524		
Platelet count	≤15.8 >15.8	1 0.526 (0.24–1.15)	0.1083	1 0.719 (0.30–1.70)	0.4517
(× 10^4^/mm^3^)					
Albumin (g/dL)	≤3.8 >3.8	1 0.733 (0.42–1.27)	0.2699		
AST (IU/L)	≤42 >42	0.871 (0.52–1.47) 1	0.6038		
ALT (IU/L)	≤39 >39	0.889 (0.55–1.45) 1	0.6367		
Tumor size (mm)	≤20 >20	0.936 (0.54–1.61) 1	0.8118		
Tumor number	Single Multiple	0.467 (0.28–0.77) 1	0.003	0.558 (0.33–0.96) 1	0.0344
AFP (ng/mL)	≤10 >10	0.575 (0.35–0.95) 1	0.0304	0.595 (0.32–1.10) 1	0.098
DCP (mAU/mL)	≤40 >40	0.720 (0.41–1.25) 1	0.244		

HBsAg, hepatitis B surface antigen; DAA, direct-acting antiviral; IFN, interferon; ALBI score, albumin–bilirubin score; AST, aspartate aminotransferase; ALT, alanine aminotransferase; AFP, α-fetoprotein; DCP, des-γ-carboxy prothrombin.

**Table 4:** Univariate and multivariate analyses of 2nd HCC recurrence-free survival after PSM.

Variable	Category	Univariate Analysis		Multivariate Analysis	
		HR (95% CI)	*p*-Value	HR (95% CI)	*p*-Value
Age	≤74 >74	1 1.546 (0.72–3.30)	0.2601		
Sex	Male Female	1 0.669 (0.31–1.44)	0.3059		
HBsAg	Positive Negative	1.4839 × 10^−8^ (0–0)	0.9991		
DAA	Untreated Treated	1 0.387 (0.18–0.84)	0.0613	1 0.424 (0.19–0.96)	0.0394
Alcohol history	+ −	1.544 (0.62–3.85) 1	0.3511		
Diabetes mellitus	+ −	1.431 (0.64–3.20) 1	0.3827		
IFN treatment history	+ −	0.648 (0.26–1.61) 1	0.3504		
Child–Pugh	5 6–7	0.422 (0.19–0.94) 1	0.0355	0.563 (0.24–1.33) 1	0.1918
mALBI grade	1–2a 2b–3	0.534 (0.25–1.15) 1	0.1075		
FIB-4 index	≤3.25 >3.25	0.704 (0.21–2.34) 1	0.5665		
Platelet count(× 10^4^/mm^3^)	≤15.8 >15.8	1 0.723 (0.25–2.09)	0.5493		
Albumin (g/dL)	≤3.8 >3.8	1 0.791 (0.35–1.81)	0.5784		
AST (IU/L)	≤42 >42	0.755 (0.32–1.79) 1	0.5234		
ALT (IU/L)	≤39 >39	1.021 (0.47–2.23) 1	0.9581		
Tumor size (mm)	≤20 >20	0.699 (0.31–1.56) 1	0.3808		
Tumor number	Single Multiple	0.307 (0.14–0.66) 1	0.0023	0.462 (0.21–1.04) 1	0.0609
AFP (ng/mL)	≤10 >10	0.378 (0.16–0.90)	0.0271	0.542 (0.21–1.40) 1	0.2064
		1			
DCP (mAU/mL)	≤40 >40	0.999 (0.40–2.48) 1	0.9994		

IFN, interferon; ALBI, albumin–bilirubin; AST, aspartate aminotransferase; ALT, alanine aminotransferase; AFP, α-fetoprotein; DCP, des-γ-carboxy prothrombin.

**Table 5:** Univariate and multivariate analyses of 1st and 2nd HCC recurrence-free survival in DAA-treated group after PSM.

Variable	Category	1st HCC Recurrence-Free Survival				2nd HCC Recurrence-Free Survival			
		Univariate Analysis		Multivariate Analysis		Univariate Analysis		Multivariate Analysis	
		HR (95% CI)	*p*-Value	HR (95% CI)	*p*-Value	HR (95% CI)	*p*-Value	HR (95% CI)	*p*-Value
Age	≤74 >74	1 1.289 (0.59–2.84)	0.5283			1 1.383 (0.40–4.74)	0.606		
Sex	Male Female	1 10.45 (0.50–2.18)	0.906			1 1.374 (0.42–4.52)	0.6007		
HBsAg	Positive Negative	5.3772 × 10^−9^ (0–0) 1	0.9991			3.9605 × 10^−8^ (0–0) 1	0.9991		
Alcohol history	+ −	0.628 (0.22–1.81) 1	1.3894			0.733 (0.16–3.42)	0.6931		
Diabetes mellitus	+ −	1.347 (0.62–2.90) 1	0.4473			1.761 (0.51–6.03) 1	0.3675		
IFN treatment history	+ −	0.760 (0.34–1.68) 1	0.4972			0.359 (0.08–1.68) 1	0.1985	0.245 (0.05–1.18) 1	0.0789
Child–Pugh	5 6–7	1.024 (0.49–2.13) 1	0.9486			1.032 (0.31–3.40) 1	0.9585		
mALBI grade	1–2a 2b-3	0.804 (0.39–1.67) 1	0.5591			0.669 (0.20–2.22) 1	0.5113		
FIB-4 index	≤3.25 >3.25	0.914 (0.35–2.40) 1	0.8554			1.118 (0.24–5.20) 1	0.887		
Platelet count(× 10^4^/mm^3^)	≤15.8 >15.8	1 0.450 (0.13–1.49)	0.1903	1 0.387 (0.12–1.30)	0.1243	1 0.671 (0.14–3.12)	0.611		
Albumin (g/dL)	≤3.8 >3.8	1 0.639 (0.28–1.44)	0.2816			1 0.605 (0.16–2.29)	0.4584		
AST (IU/L)	≤42 >42	0.964 (0.44–2.12) 1	0.9261			0.770 (0.20–2.91) 1	0.7004		
ALT (IU/L)	≤39 >39	1.111 (0.52–2.35) 1	0.7839			0.625 (0.17–2.35) 1	0.4882		
Tumor size (mm)	≤20 >20	1.104 (0.45–2.72) 1	0.8294			1.689 (0.36–7.82) 1	0.5032		
Tumor number	Single Multiple	0.540 (0.24–1.22) 1	0.1388	0.469 (0.20–1.07) 1	0.0731	0.180 (0.05–0.60) 1	0.0051	0.141 (0.04–0.48) 1	0.0016
AFP (ng/mL)	≤10 >10	0.670 (0.31–1.44) 1	0.3066			0.454 (0.12–1.72) 1	0.2451		
DCP (mAU/mL)	≤40 >40	0.788 (0.34–1.85) 1	0.5839			1.750 (0.38–8.14) 1	0.4755		

HBsAg, hepatitis B surface antigen; DAA, direct-acting antiviral; IFN, interferon; ALBI, albumin–bilirubin; AST, aspartate aminotransferase; ALT, alanine aminotransferase; AFP, α-fetoprotein; DCP, des-γ-carboxy prothrombin.

## Data Availability

Data that support the findings of this study are available from the author, R.K. (Ryoko Kuromatsu), on reasonable request.

## Supplementary Materials

The following supporting information can be downloaded at: https://www.mdpi.com/article/10.3390/cancers14092295/s1, Figure S1: Study design; Figure S2: First recurrence rate after propensity score matching (PSM) analysis.; Figure S3: Recurrence and second recurrence rates with and without single tumor following HCC.
