# Supplementary material for: Hepatitis C Virus Elimination Using Direct Acting Antivirals after the Radical Cure of Hepatocellular Carcinoma Suppresses the Recurrence of the Cancer
Source: Cancers (Basel). 2022 May 4;14(9):2295. doi: 10.3390/cancers14092295 (PMC9103530; doi:10.3390/cancers14092295)
Supplement: Supplementary file 1 [file cancers-14-02295-s001.zip › cancers-1711918-Supplementary Materials.pdf]

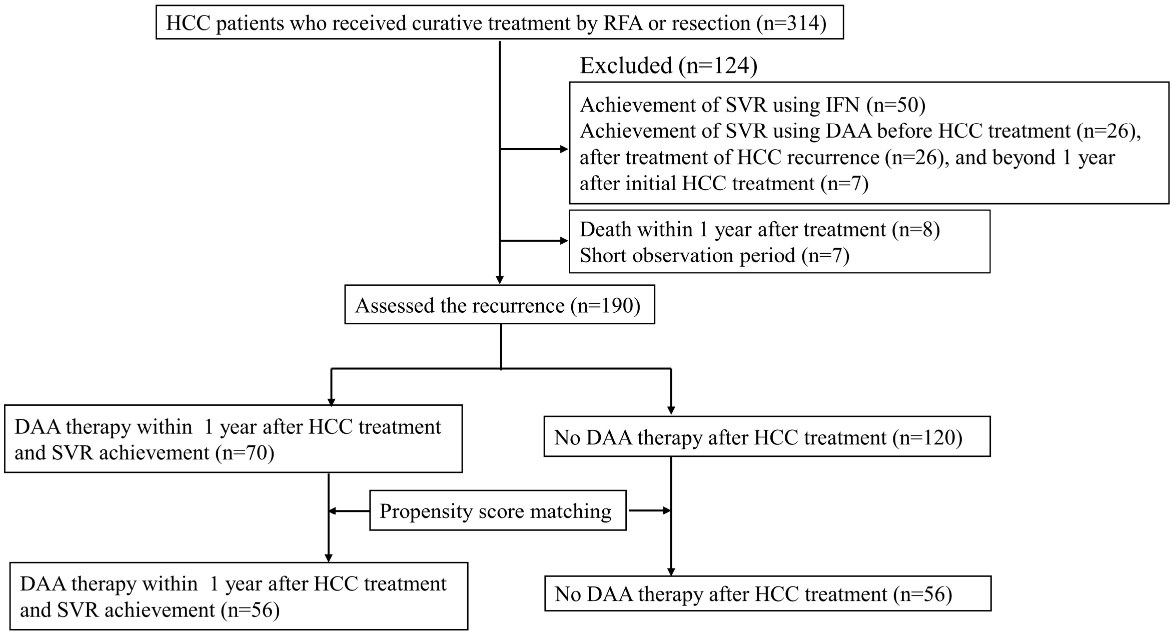

Supplementary figure S1. Study design.

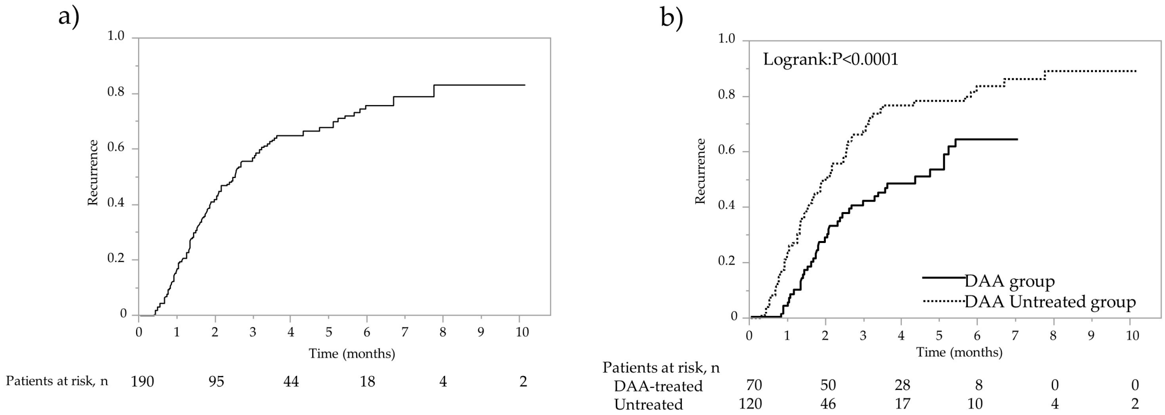

Supplementary figure S2. Recurrence rate before propensity score matching (PSM) analysis. (**a**) Overall recurrence rate of patients after curative treatment for HCC, (**b**) Recurrence rates of patients with and without DAA treatment following HCC curative treatment. Solid line represents DAA therapy group; the dotted line represents DAA untreated group.

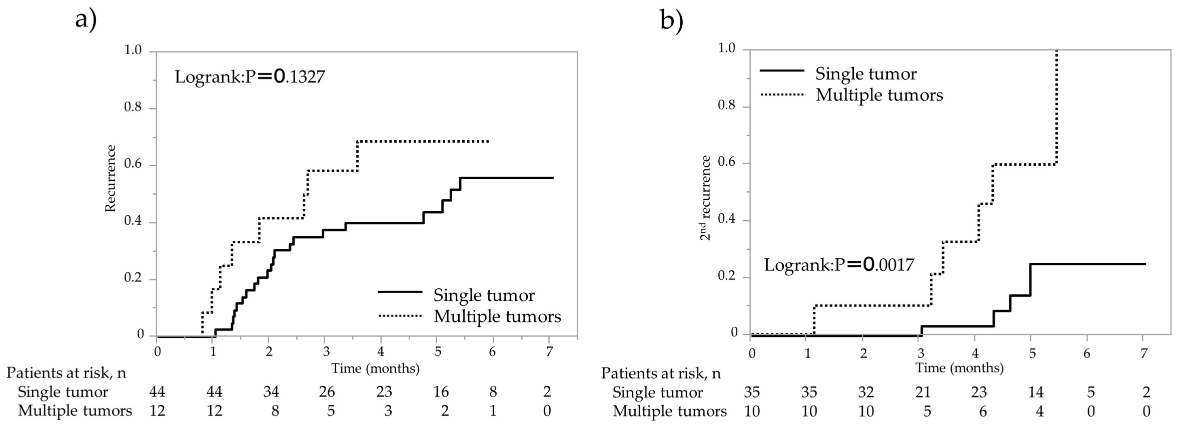

Supplementary figure S3. Recurrence and second recurrence rates with and without single tumor following HCC curative treatment in the DAA-treated group after propensity score matching (PSM) analysis. (**a**) Recurrence rate of 56 patients following HCC curative treatment, (**b**) Second recurrence rates of 45 patients with curative treatment for initial and first recurrent HCC.
